# Urinary extracellular vesicle metabolomic profiling reveals a distinct molecular signature for the non-invasive diagnosis of lupus nephritis

**DOI:** 10.3389/fimmu.2026.1741455

**Published:** 2026-02-17

**Authors:** Nan Zhang, Ning Dong, Anran Xie, Wenjing Liu, Adeel Khan, Yanjing Rui, Ping Yang

**Affiliations:** 1 Department of Laboratory Medicine, Nanjing Drum Tower Hospital, Affiliated Hospital of Medical School, Nanjing University, Nanjing, China; 2 Department of Biotechnology, University of Science and Technology Bannu, Bannu, Pakistan; 3 Department of Obstetrics and Gynecology, Nanjing Drum Tower Hospital, Affiliated Hospital of Medical School, Nanjing University, Nanjing, China; 4 State Key Laboratory of Pharmaceutical Biotechnology, Jiangsu Engineering Research Center for MicroRNA Biology and Biotechnology, NJU Advanced Institute of Life Sciences (NAILS), Nanjing University, Nanjing, China

**Keywords:** diagnostic biomarkers, lupus nephritis, metabolomic profiling, ROC curve, urinary extracellular vesicles

## Abstract

**Background:**

Lupus nephritis (LN) is a severe complication of systemic lupus erythematosus (SLE), underscoring an urgent need for non-invasive diagnostic biomarkers.

**Objective:**

This study aimed to define the metabolomic signature of urinary extracellular vesicles (uEVs) in LN and to identify novel biomarkers for precision diagnosis.

**Methods:**

uEVs were isolated from urine samples of 29 SLE patients with LN, 22 SLE patients without renal involvement, and 20 healthy controls (HCs) using a standardized precipitation-based protocol. uEVs were rigorously characterized in accordance with the Minimal Information for Studies of Extracellular Vesicles (MISEV) guidelines, including transmission electron microscopy, nanoparticle tracking analysis, and the assessment of canonical EV markers. Comprehensive untargeted metabolomic profiling of uEVs was subsequently performed using liquid chromatography–tandem mass spectrometry (LC–MS/MS).

**Results:**

A total of 284 differential metabolites were identified between LN patients and the SLE group, including 230 upregulated and 54 downregulated metabolites. Machine learning–based feature prioritization using a random forest algorithm identified a panel of ten candidate metabolites. Notably, three metabolites—Glucosylsphingosine (Lyso-Gb1), phosphatidylethanolamine N-methylated (PE-NMe), and PC(20:5/TXB2)—demonstrated excellent discriminatory performance for differentiating LN from non-renal SLE, with areas under the receiver operating characteristic curve (AUCs) of 0.912, 0.906, and 0.897, respectively.

**Conclusion:**

We identified a distinct uEV metabolic signature in LN and developed a robust, non-invasive biomarker panel. This strategy holds significant promise for the early detection and personalized management of LN, offering a compelling alternative to invasive renal biopsy.

## Introduction

SLE is a complex autoimmune disorder characterized by widespread immune dysregulation, leading to autoantibody production and multi-organ injury (1–5). Among its most severe complications is LN, which develops in up to 40% of patients and is a primary determinant of morbidity and mortality. Despite advances in therapy, 5–20% of LN patients progress to end-stage renal disease within a decade, highlighting the critical need for early and accurate diagnosis (6–8). However, this goal remains challenging: LN onset is often insidious, conventional urinalysis lacks sensitivity, and the diagnostic gold standard of renal biopsy—an invasive procedure—is ill-suited for repeated monitoring (9, 10). Consequently, there is an urgent, unmet need for reliable non-invasive biomarkers to enable timely detection and guide personalized treatment (11).

Liquid biopsy has emerged as a promising approach for non-invasive disease assessment. Extracellular vesicles (EVs), which are secreted into biofluids, have garnered significant interest as rich reservoirs of proteins, lipids, and nucleic acids that mirror the pathophysiological state of their parental cells (12–14). Urinary EVs (uEVs) are of particular value in nephrology, as they provide a concentrated source of kidney-derived molecules and offer a window into renal pathology that is obscured in whole urine analyses (15–17). While previous studies, including our own, have extensively explored the proteomic and transcriptomic cargo of EVs (18–23), the uEV metabolome—a dynamic readout of cellular activity and metabolic reprogramming in autoimmunity—has been largely overlooked.

We hypothesized that the uEV metabolome encapsulates the renal-specific metabolic disturbances inherent to LN. Here, we performed high-resolution untargeted metabolomic profiling of uEVs from the SLE with LN, SLE without LN, and HC groups. By integrating bioinformatics with random forest machine learning, we identified and validated a distinct metabolic signature of LN, revealing a panel of biomarkers with high diagnostic accuracy. Our findings underscore uEV metabolomics as a powerful, non-invasive approach for the precision diagnosis and stratification of LN.

## Materials and methods

### Patient and control enrollment

**Figure 1:**
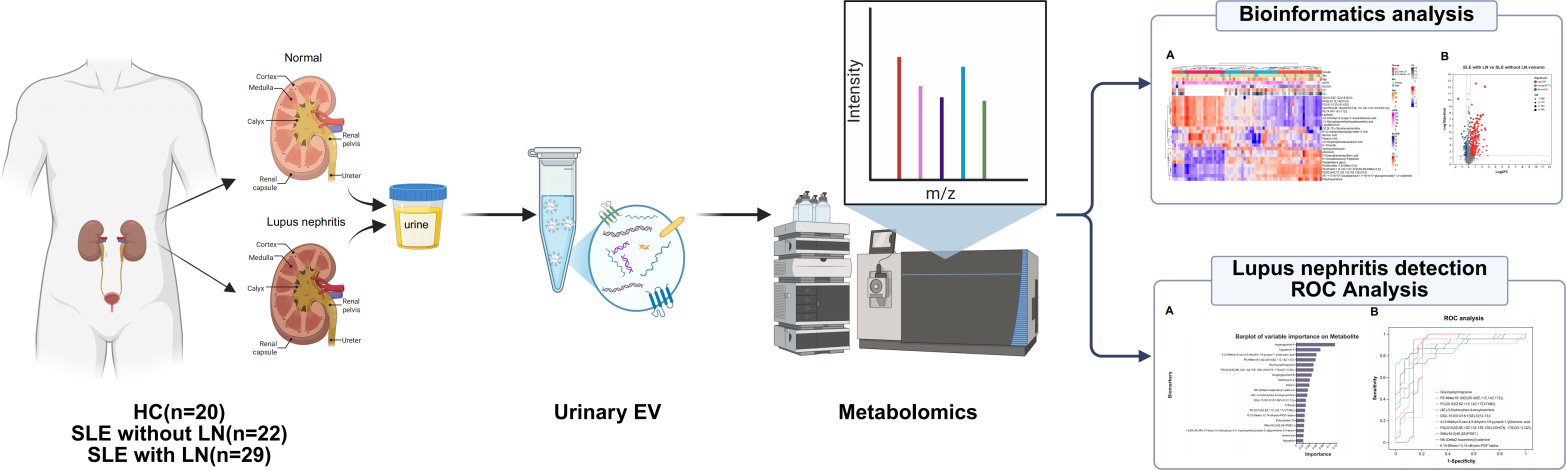
Study workflow overview. Urine samples were collected from healthy controls (HC, n=20) and SLE patients stratified by the presence of lupus nephritis (non-LN, n=22; LN, n=29) for uEV isolation. Isolated uEVs underwent untargeted metabolomic profiling, followed by comprehensive bioinformatics analysis and machine learning–based feature selection (including variable importance and ROC analysis) to identify and validate distinct metabolic signatures for LN diagnosis.

Patients with SLE were enrolled according to the 2019 EULAR/ACR classification criteria, ensuring diagnostic consistency across the study cohort (24). LN was defined as renal involvement in SLE patients, either by a 24-hour urinary protein excretion ≥500 mg or according to the International Society of Nephrology/Renal Pathology Society (ISN/RPS) classification criteria, with histopathological evaluation based on the 2018 revised ISN/RPS classification for LN (25, 26). HCs were age- and sex-matched volunteers with no history of autoimmune or chronic disease, normal laboratory tests, and no structural abnormalities on imaging (chest CT, abdominal and urinary system ultrasound). Individuals with prior severe infection, malignancy, metabolic or autoimmune disorders, or other conditions affecting renal function were excluded. The detailed flowchart of participant enrollment and study design is illustrated in **Figure 1**.

### Study population and sample collection

Between March 2022 and March 2024, 51 SLE patients were enrolled from the Department of Rheumatology and Immunology, Nanjing Drum Tower Hospital, Affiliated Hospital of Medical School, Nanjing University. Patients were stratified into SLE with LN (*n* = 29) and SLE without LN (*n* = 22) subgroups according to renal involvement. Twenty age- and sex-matched HCs were recruited from the hospital’s Physical Examination Center. Demographic characteristics, clinical data, and treatment histories were systematically recorded. After an overnight fast (≥8 h), midstream morning urine samples (~8 mL) were collected under sterile conditions. The samples were centrifuged at 2,000 × g for 30 min at room temperature to remove cellular debris, and the resulting supernatants were aliquoted into 2-mL tubes and stored at −80 °C until further analysis.

### Isolation and characterization of uEVs

The uEVs were isolated using a commercial precipitation-based kit (Total Exosome Isolation Reagent; Thermo Fisher Scientific, USA) according to the manufacturer’s instructions. Briefly, thawed urine samples were centrifuged at 2,000 × g for 30 min at 4 °C to remove residual debris. Subsequently, 800 µL of the clarified supernatant was mixed with an equal volume of the isolation reagent, vortexed thoroughly, and incubated at room temperature for 1 h. The mixture was then centrifuged at 10,000 × g for 1 h at 4 °C, and the resulting uEV pellet was resuspended in sterile phosphate-buffered saline (PBS) and stored at −80 °C until further analysis. The morphology of uEVs was examined by transmission electron microscopy (TEM) following negative staining with 2% phosphotungstic acid. Particle size distribution and concentration were quantified using nanoparticle tracking analysis (NTA; NanoSight, UK), with sample dilutions adjusted to achieve approximately 200 particles per field. The expression of canonical uEV protein markers was verified by Western blotting, performed using standard SDS–PAGE and PVDF membrane transfer procedures.

### Metabolite extraction

Metabolites were extracted from uEV suspensions using a protein precipitation procedure. Briefly, 100 µL of each uEV suspension was mixed with 400 µL of ice-cold acetonitrile/methanol (1:1, v/v) containing L-2-chlorophenylalanine (0.02 mg/mL) as an internal standard. The mixture was vortexed for 30 s and subjected to ultrasonic extraction (5 °C, 40 kHz) for 30 min. Samples were then incubated at −20 °C for 30 min to facilitate protein precipitation, followed by centrifugation at 13,000 × g for 15 min at 4 °C. The resulting supernatants were evaporated to dryness under a gentle nitrogen stream, reconstituted in 100 µL of acetonitrile/water (1:1, v/v), sonicated for 5 min, and centrifuged again (13,000 × g, 10 min, 4 °C). The clarified supernatants were transferred to LC–MS vials for subsequent metabolomic analysis. Quality control (QC) samples were prepared by pooling equal aliquots from all study samples and injected periodically (one QC sample per 5 study samples) throughout the analytical sequence to monitor instrument stability and assess analytical reproducibility.

### LC–MS/MS analysis

Untargeted metabolomic profiling was performed using an ultrahigh-performance liquid chromatography (UHPLC) system coupled to a Q Exactive™ Exploris 240 mass spectrometer (Thermo Fisher Scientific, USA). Chromatographic separation was achieved on an ACQUITY UPLC HSS T3 column (100 × 2.1 mm, 1.8 μm; Waters, USA) maintained at 40 °C, with an injection volume of 10 μL. The mobile phase consisted of (A) 0.1% formic acid in water/acetonitrile (95:5, v/v) and (B) acetonitrile/isopropanol/water (47.5:47.5:5, v/v/v) containing 0.1% formic acid. Mass spectrometric detection was performed in both positive and negative electrospray ionization (ESI) modes over an m/z range of 70–1050. The optimized ion source parameters were as follows: sheath gas pressure, 60 psi; auxiliary gas pressure, 20 psi; auxiliary gas heater temperature, 350 °C; capillary temperature, 320 °C; and spray voltages of +3.4 kV and −3.0 kV for the respective modes. Data were acquired in data-dependent acquisition (DDA) mode, with full MS and MS/MS resolutions of 60,000 and 15,000, respectively. Higher-energy collisional dissociation (HCD) was applied with stepped collision energies of 20, 40, and 60 eV.

### Data processing and statistical analysis

For metabolomics analysis, raw LC–MS/MS data were processed using Progenesis QI software (Waters, USA) for alignment, peak detection, and annotation against HMDB and Metlin databases, achieving metabolite identification at MSI Level 2 confidence. Features detected in >80% of samples were retained, missing values were imputed (group minimum), and signal intensities were normalized to total ion current; variables with >30% RSD in QC samples were removed. Multivariate analyses (PCA and OPLS-DA) were performed using the ropls R package, with model robustness validated by seven-fold cross-validation and a permutation test (n = 200). Differential metabolites were defined by a VIP score ≥ 1.0, fold change (FC) ≥ 1.2 (or ≤ 0.83), and an P-value < 0.05. Pathway enrichment was conducted using the KEGG database.

For clinical and general statistical comparisons, data were analyzed using SPSS 24.0 (IBM Corp., USA). Continuous variables were tested for normality (Shapiro–Wilk test) and presented as mean ± SD (analyzed by Student’s t-test) or median with IQR (analyzed by Mann–Whitney U test). These univariate tests were also applied to the metabolomics data to derive P-values. Categorical variables were compared using the Chi-square or Fisher’s exact test. A two-tailed P-value < 0.05 was considered significant. Data visualization was performed using GraphPad Prism 8.0, GenesCloud (for heatmaps/volcano plots), and BioRender (for schematics).

## Results

### Characteristics of study participants and laboratory parameters

A total of 51 patients with SLE were enrolled, including 29 patients with LN and 22 patients without renal involvement, alongside 20 age- and sex-matched HCs. All LN patients tested positive for antinuclear antibodies, and 24-hour urinary protein excretion exceeded 500 mg, consistent with established diagnostic criteria for LN. Additional laboratory parameters—including routine hematological tests, renal function indices, and immunological markers—are summarized in **Table 1**.

**Table 1:** Baseline demographic and clinical characteristics of included individuals.

Variables	HC(N = 20)	SLE without LN (N = 22)	SLE with LN (N = 29)
Gender (n, %)			
Male	4 (20.0)	3 (13.6)	5 (17.2)
Female	16 (80.0)	19 (86.4)	24 (82.8)
Age (year)			
Mean ± SD	34.3 ± 11.3	36.3 ± 15.7	32.9 ± 12.7
Median (IQR)	31.0 (24.5 ˜ 42.0)	35.0 (21.0 ˜ 48.8)	33.0 (23.8 ˜ 47.8)
Range	21.0 ˜ 55.0	13.0 ˜ 62.0	15.0 ˜ 57.0
The minimum age of illness		27.5 (15.7 ˜ 33.2)	22.0 (15.5 ˜ 30.5)
Disease duration (years)			
Median (IQR)		5.5 (0.2 ˜ 15.2)	8.0 (3.0 ˜ 11.0)
Disease activity, Median (IQR)			
SLEDAI		4.0 (2.7 ˜ 5.0)	12.0 (7.5 ˜ 17.5)
Renal SLEDAI		0.0 (0.0 ˜ 0.0)	8.0 (4.0 ˜ 10.0)
Extra-renal SLEDAl		4.0 (2.7 ˜ 5.0)	5.0 (3.0 ˜ 9.0)
Organ involvement (n, %)			
Mucocutaneous involvement		12 (54.5)	9 (31.0)
Musculoskeletal involvement		6 (27.3)	3 (10.3)
Serositis		0 (0.0)	0 (0.0)
Neuropsychiatric involvement		0 (0.0)	1 (3.4)
Pulmonary involvement		2 (9.1)	8 (27.6)
Renal involvement		0 (0.0)	29 (100.0)
Cardiovascular involvement		6 (27.3)	4 (13.8)
Hematological		8 (36.4)	22 (75.9)
Laboratory assessment, Median (IQR)			
24h-UP (mg/24h)		298.5 (153.8 ˜ 352.5)	2783.0 (1738.5 ˜ 6355.5)
ACR (mg/g)		23.6 (9.5 ˜ 64.2)	1398.4 (576.6 ˜ 2630.0)
eGFR (mL/min/1.73m^2^)		128.0 (95.9 ˜ 150.3)	73.5 (37.5 ˜ 123.5)
Casts (N/μL)	0 (0 ˜ 1.0)	0 (0 ˜ 0.3)	1.0 (0 ˜ 2.0)
WBC (10^9^/L)	5.4 (4.9 ˜ 7.3)	4.6 (3.3 ˜ 5.7)	4.7 (3.6 ˜ 6.9)
Lymphocytes (10^9^/L)	1.9 (1.7 ˜ 2.2)	1.2 (0.5 ˜ 1.5)	0.9 (0.5 ˜ 1.5)
ESR (mm/h)		9.5 (7.0 ˜ 31.0)	46.5 (29.8 ˜ 69.3)
GLU (mmol/L)	5.0 (4.9 ˜ 5.4)	4.2 (3.8 ˜ 4.9)	4.3 (3.8 ˜ 4.9)
UREA (mmol/L)	4.5 (3.9 ˜ 5.3)	4.6 (4.0 ˜ 6.8)	8.2 (5.4 ˜ 16.4)
CREA (µmol/L)	56.0 (48.3 ˜ 63.8)	55.0 (42.8 ˜ 61.8)	86.0 (53.0 ˜ 159.0)
VD (ng/mL)		13.1 (11.5 ˜ 19.9)	10.2 (6.6 ˜ 19.1)
C3 (g/L)		0.68 (0.61 ˜ 1.07)	0.73 (0.52 ˜ 0.83)
C4 (g/L)		0.12 (0.09 ˜ 0.26)	0.15 (0.09 ˜ 0.24)
Anti-dsDNA (IU/mL)		110.2(15.0 ~ 466.4)	189.9 (107.9 ~ 529.6)
ANA (percentage)		(+): 93.8%	(+): 100.0%
ASMA (percentage)		(+): 30.8%	(+): 43.8%
aB2GP1 (RU/ml)		8.4 (5.8 ˜ 10.4)	9.4 (6.2 ˜ 17.8)
ACA-IgG (GPL-U/ml)		1.3 (0.8 ˜ 5.0)	0.6 (0.0 ˜ 5.0)
ACA-IgM (MPL-U/ml)		1.0 (0.8 ˜ 1.9)	1.9 (1.4 ˜ 2.7)
ACA-IgA/G/M (RU/ml)		0.95 (0.0 ˜ 7.8)	3.8 (1.2 ˜ 10.2)
Renal histology-ISN/RPS class, n(%)			
III			2 (6.9)
III+IV			2 (6.9)
IV			1 (3.4)
IV+V			3 (10.3)
V			1 (3.4)
Treatment (n, %)			
Prednisone		15 (68.2)	19 (65.5)
Methylprednisolone		5 (22.7)	9 (31.0)
Hydroxychloroquine		14 (63.6)	14 (48.3)
Cyclophosphamide		0 (0.0)	5 (17.2)
Tacrolimus		5 (22.7)	10 (34.5)
Mycophenolate mofetil		1 (4.5)	9 (31.0)
Leflunomide		2 (9.1)	5 (17.2)
Azathioprine		2 (9.1)	1 (3.4)
Methotrexate		2 (9.1)	1 (3.4)
Belimumab		5 (22.7)	2 (6.9)

SD, standard deviation; IQR, interquartile range. HC healthy control; SLE systemic lupus erythematosus; LN, lupus nephritis; SLE-DAI systemic lupus erythematosus disease activity index; 24h-UP 24-hour urinary protein; ACR urinary protein/creatinine ratio; WBC white blood cell; ESR erythrocyte sedimentation rate; GLU glucose; UREA carbamide; CREA, creatinine; VD vitamin D; Anti-dsDNA anti-double stranded DNA; ANA antinuclear antibody; ASMA anti-smooth muscle antibody; aB2GP1 anti-beta-2-glycoprotein 1 antibody; ACA-IgG anti cardiolipin antibody immunoglobulin G; ACA-IgM anti cardiolipin antibody immunoglobulin M; ACA-IgA anti cardiolipin antibody immunoglobulin A.

### uEVs are successfully isolated and exhibit typical characteristics

**Figure 2:**
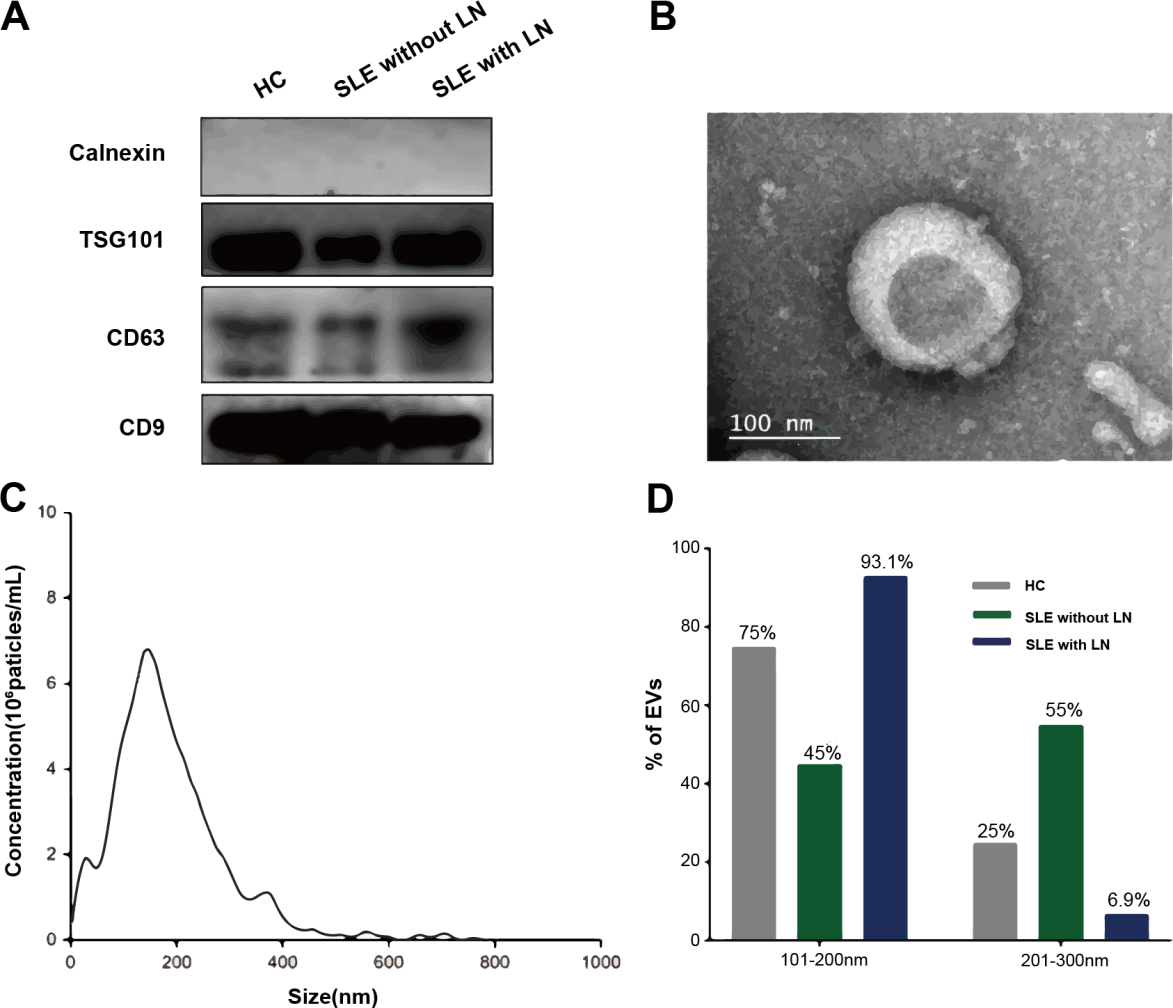
Characterization of uEVs. **(A)** Western blot analysis showing the enrichment of canonical EV markers. **(B)** Representative TEM images illustrating the typical cup-shaped morphology of uEVs. **(C)** NTA depicting the size distribution of EV particles. **(D)** Quantitative histogram comparing EV size distributions among the three groups: SLE without LN, SLE with LN, and HC.

The uEVs were isolated and characterized following the Minimal Information for Studies of Extracellular Vesicles (MISEV) 2018 guidelines. Western blot analysis confirmed the enrichment of canonicalEV markers (TSG101, CD63, CD9) and the absence of the endoplasmic reticulum protein calnexin, indicating minimal cellular contamination (**Figure 2A**). TEM revealed the typical cup-shaped morphology and bilayered membrane structure of EVs (**Figure 2B**). NTA demonstrated a unimodal size distribution with a mean diameter of approximately 120 nm (**Figures 2C, D**). Collectively, these results confirm the successful isolation of high-purity uEVs, suitable for subsequent metabolomic profiling.

### The uEV metabolome distinguishes SLE with LN from SLE without LN and HC groups

**Figure 3:**
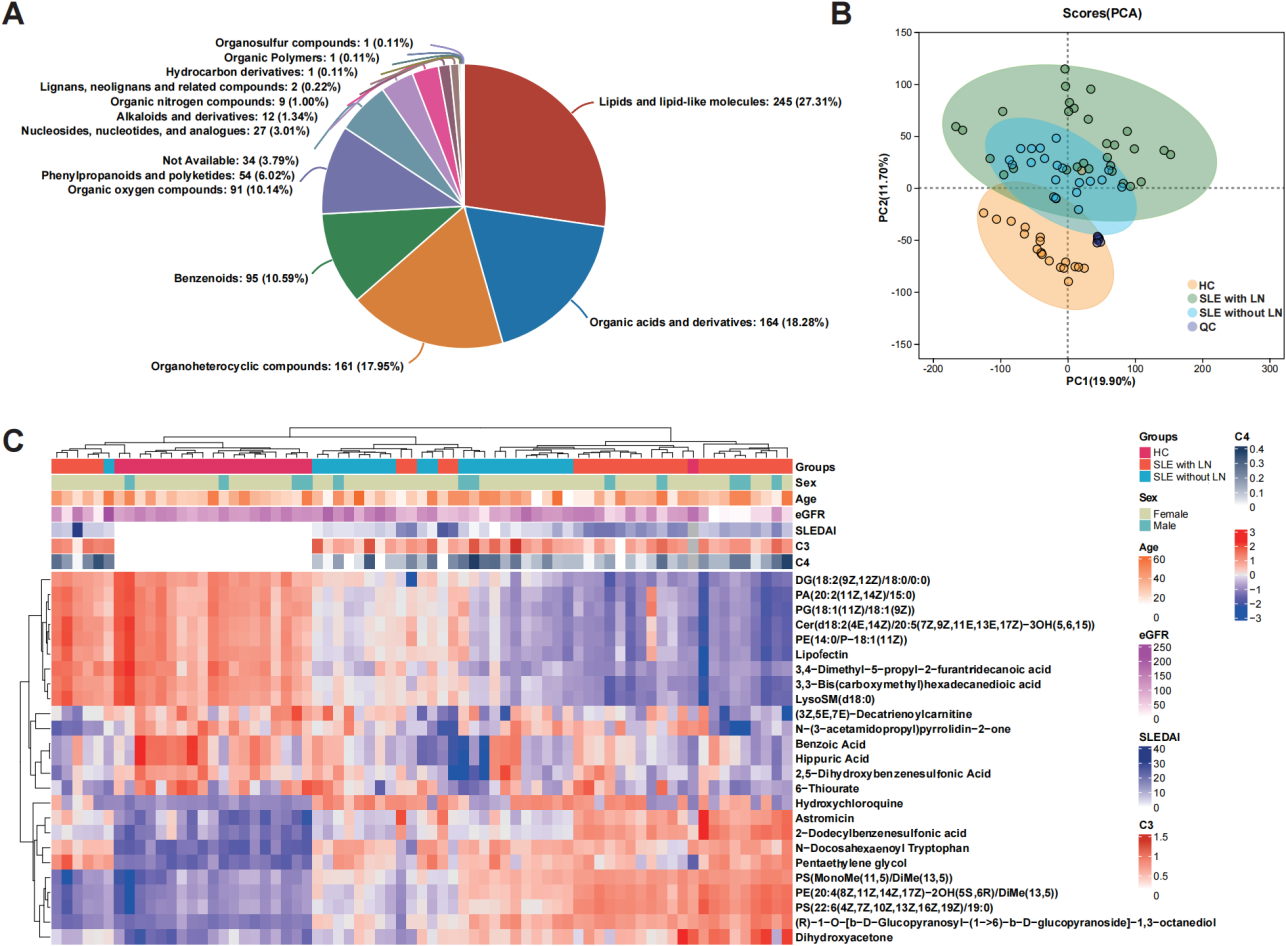
Metabolic profiling of uEVs across disease and control groups. **(A)** Classification of metabolites identified from uEV samples according to chemical taxonomy. **(B)** PCA score plot illustrating global metabolic variation and separation among the SLE without LN, SLE with LN, and HC groups. **(C)** Hierarchical clustering heatmap showing the relative abundance patterns of commonly detected metabolites across the HC, SLE without LN, and SLE with LN groups.

Untargeted metabolomic profiling of uEVs identified metabolites spanning multiple chemical classes, with lipid-associated species representing the dominant category across all samples (**Figure 3A**). PCA demonstrated a progressive shift in the global uEV metabolic landscape from HCs to SLE patients without LN and to LN patients, indicating disease-associated metabolic remodeling (**Figure 3B**). While most LN samples formed a distinct cluster separated from HC, a subset of LN patients clustered closely with HC in the PCA space, showing partial overlap of uEV metabolic profiles between these individuals and healthy subjects. Hierarchical clustering analysis further confirmed this pattern, as several LN samples were grouped together with HC rather than with the main LN cluster based on their metabolite abundance profiles (**Figure 3C**). This observation suggests that uEV metabolic alterations in LN are heterogeneous and may be influenced by factors such as differences in renal disease activity, treatment exposure, or partial metabolic normalization in certain LN patients, resulting in uEV profiles that more closely resemble those of healthy controls.

### Identification of differential metabolites associated with LN

**Figure 4:**
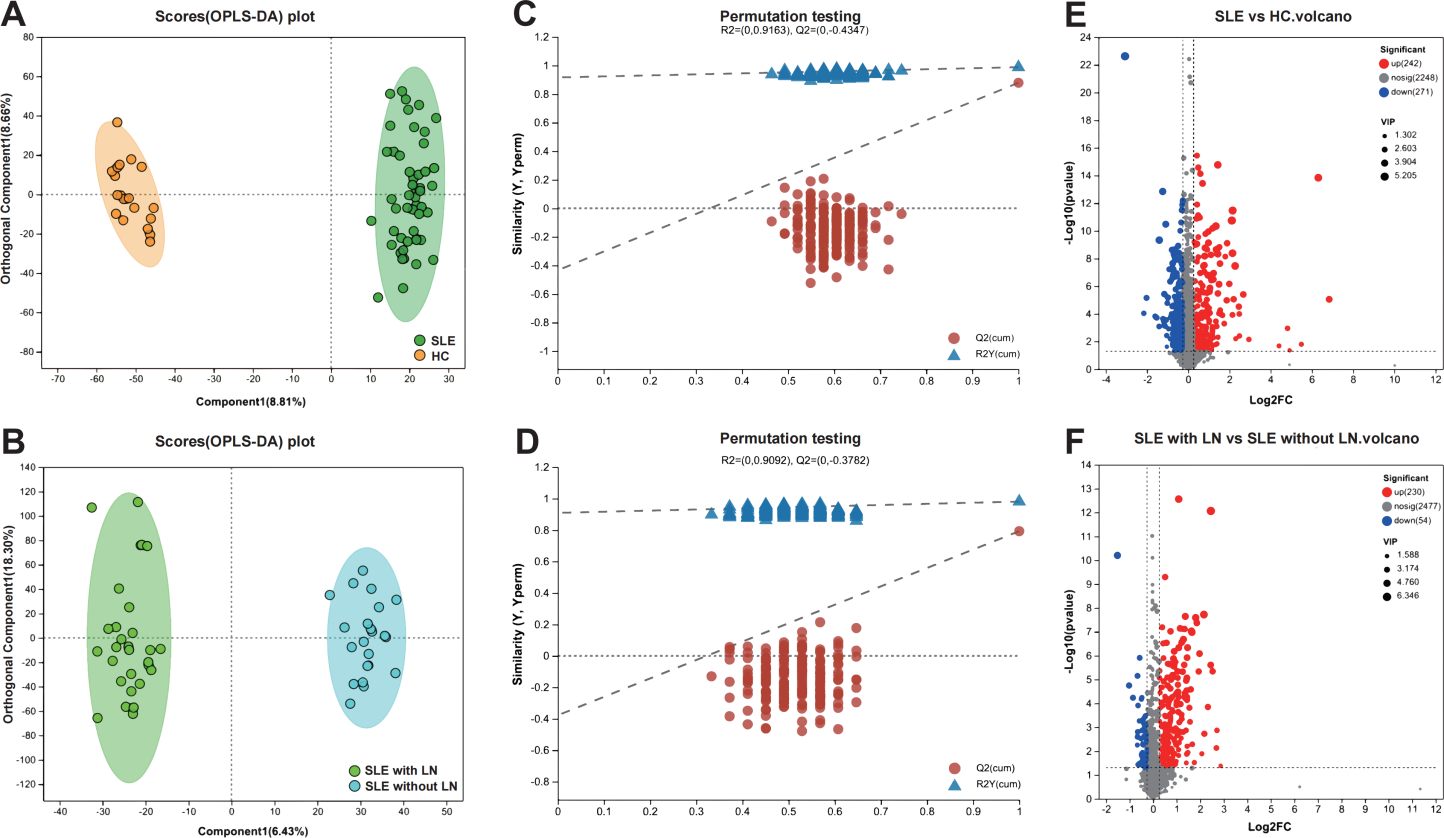
Screening and visualization of differential uEV metabolites. **(A, B)** Principal component analysis (PCA) and OPLS-DA score plots illustrating group separation among study cohorts. **(C, D)** OPLS-DA permutation test plots validating the robustness and predictive performance of the discriminant models. **(E, F)** Volcano plots highlighting significantly dysregulated metabolites between groups, based on VIP scores and adjusted *P* values.

Multivariate statistical analyses were performed in SIMCA-P to compare metabolomic profiles between the total SLE cohort and HCs, as well as between the SLE with LN and SLE without LN subgroups. OPLS-DA score plots in mixed ion mode (ESI^+^/ESI^-^ combined) revealed clear separation between groups, indicating distinct metabolic phenotypes (**Figures 4A, B**). Model validity was further confirmed by 200-permutation tests, demonstrating robust predictive performance (**Figures 4C, D**). Differential metabolites were identified based on VIP > 1, fold change (FC) > 1.2, and P < 0.05. A total of 513 differential metabolites were detected between SLE patients and HCs (242 upregulated, 271 downregulated), while 284 differential metabolites were identified between LN and non-renal SLE subgroups (230 upregulated, 54 downregulated) (**Figures 4E, F**). The analysis successfully identified and validated distinct and reliable differences in the uEV metabolomic profiles among the SLE with LN, SLE without LN, and HC groups.

### Metabolic pathway enrichment implicates sphingolipid and phospholipid remodeling in LN

**Figure 5:**
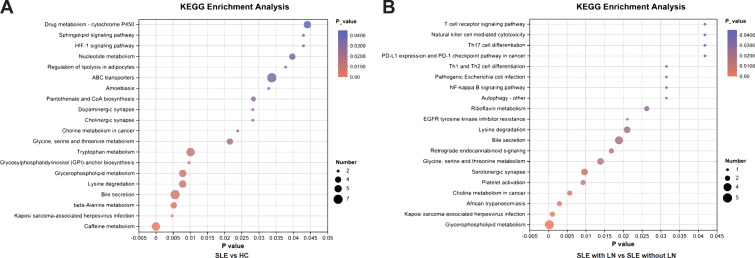
KEGG pathway enrichment analysis of differential uEV metabolites. **(A)** Enriched KEGG pathways identified by comparing SLE patients with HCs, highlighting metabolic pathways perturbed during disease onset. Pathway significance is based on nominal (unadjusted) P values derived from KEGG enrichment analysis of the predefined differential metabolites. **(B)** Enriched KEGG pathways identified by comparing LN patients with SLE patients without renal involvement, revealing pathways specifically associated with renal involvement. Enrichment results are reported using nominal (unadjusted) P values rather than false discovery rate–adjusted values, as the analysis was hypothesis-driven and focused on selected metabolites of interest.

To further explore the biological functions of the identified differential metabolites, we performed pathway enrichment analysis using the KEGG database. As visualized in the bubble plots (**Figures 5A, B**), the analysis revealed distinct metabolic signatures associated with SLE and lupus nephritis. Notably, lipid metabolism pathways were prominently dysregulated in both comparisons. Specifically, Sphingolipid metabolism and Glycerophospholipid metabolism emerged as the top-enriched pathways, exhibiting high statistical significance and metabolite counts. Additionally, pathways such as Linoleic acid metabolism and Purine metabolism were also significantly altered, particularly in the stratification of LN patients. These findings suggest that the profound remodeling of lipid and nucleotide metabolism may drive the pathogenesis of SLE and its renal complications. Detailed enrichment results are listed in **Supplementary Tables 1**, **2**.

### A random forest model identifies a diagnostic metabolite panel for LN

**Figure 6:**
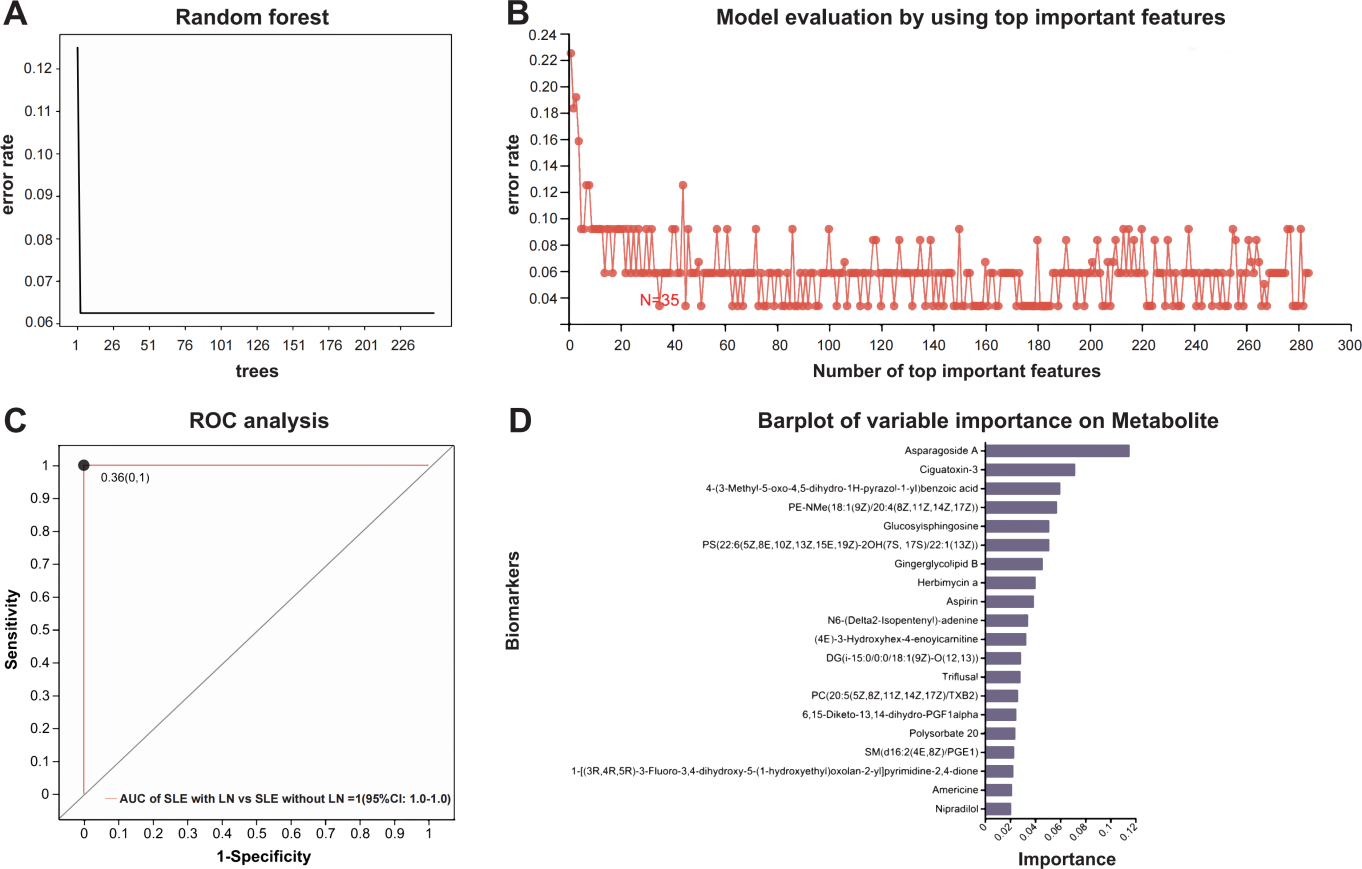
Construction and evaluation of the Random Forest model. **(A)** Error rate trend during decision tree optimization. **(B)** Feature selection and model evaluation using RFECV (Recursive Feature Elimination with Cross-Validation). **(C)** ROC curve for model validation; the curve represents the dichotomous model, points indicate optimal cut-off values, with coordinates shown in parentheses. **(D)** Importance ranking of candidate metabolites.

To differentiate SLE with LN from SLE without LN, we constructed a Random Forest (RF) classifier. We first validated the model structure, where the classification error stabilized with increasing tree numbers, ensuring model stability and mitigating overfitting (**Figure 6A**). Subsequently, Recursive Feature Elimination with Cross-Validation (RFECV) was employed to optimize the feature set, identifying a panel of 35 priority metabolites (**Supplementary Table 3**) that yielded the lowest prediction error (**Figure 6B**). The final model demonstrated exceptional performance, achieving an Area Under the Curve (AUC) of 1.000 (95% CI: 1.000–1.000) with an optimal cut-off value of 0.36 (**Figure 6C**). Variable importance analysis (**Figure 6D**) further highlighted PE-NMe (18:1 (9Z)/20:4 (8Z,11Z,14Z,17Z)), Lyso-Gb1, and PC(20:5 (5Z,8Z,11Z,14Z,17Z)/TXB2) as the top discriminators. These findings establish a robust, non-invasive diagnostic tool for LN, underscoring the potential of specific lipid metabolic signatures in disease stratification.

### Comparison of uEV metabolomic signatures with conventional clinical biomarkers

**Figure 7:**
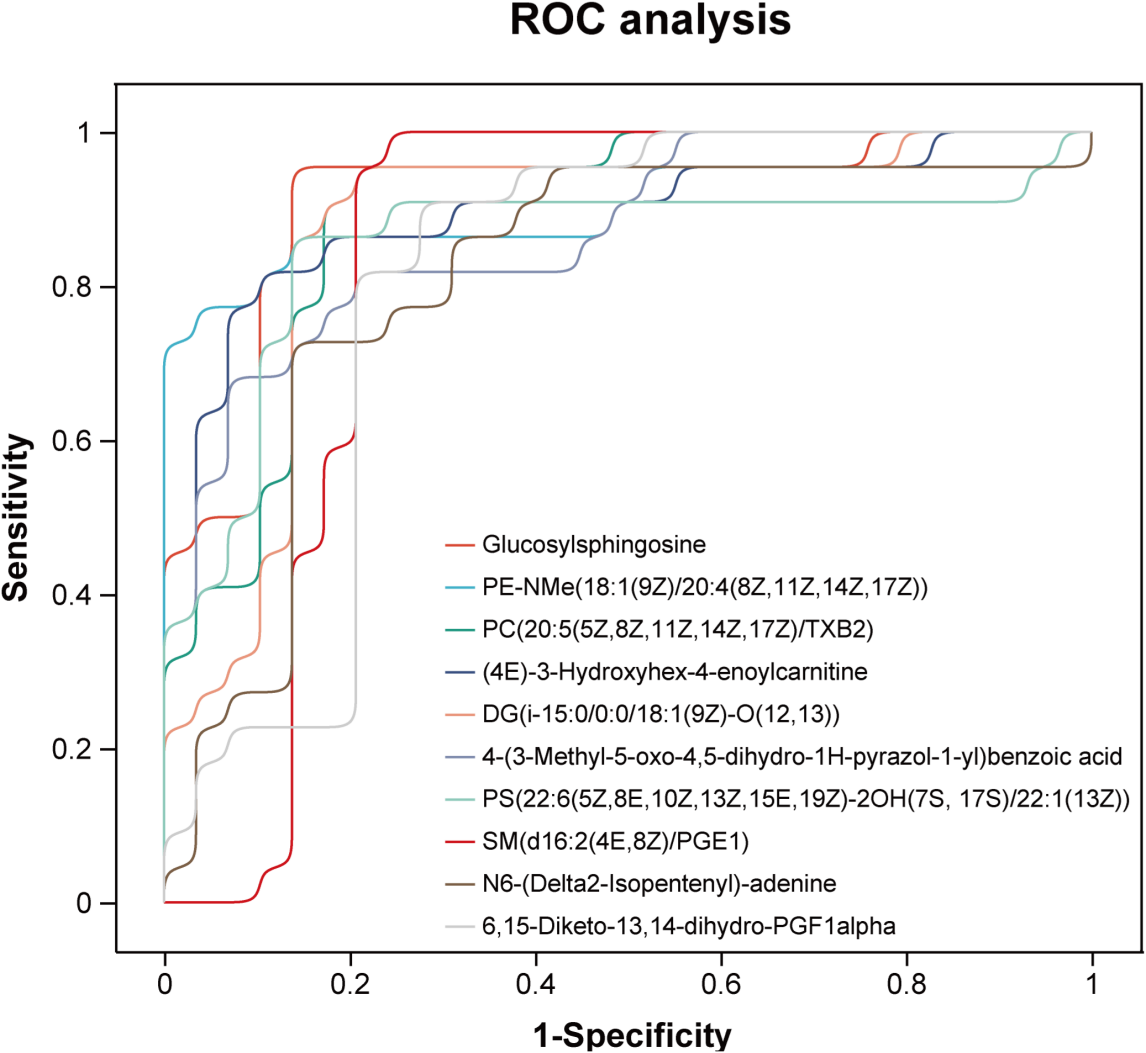
Receiver operating characteristic (ROC) curves of metabolites with potential diagnostic value for LN.

**Figure 8:**
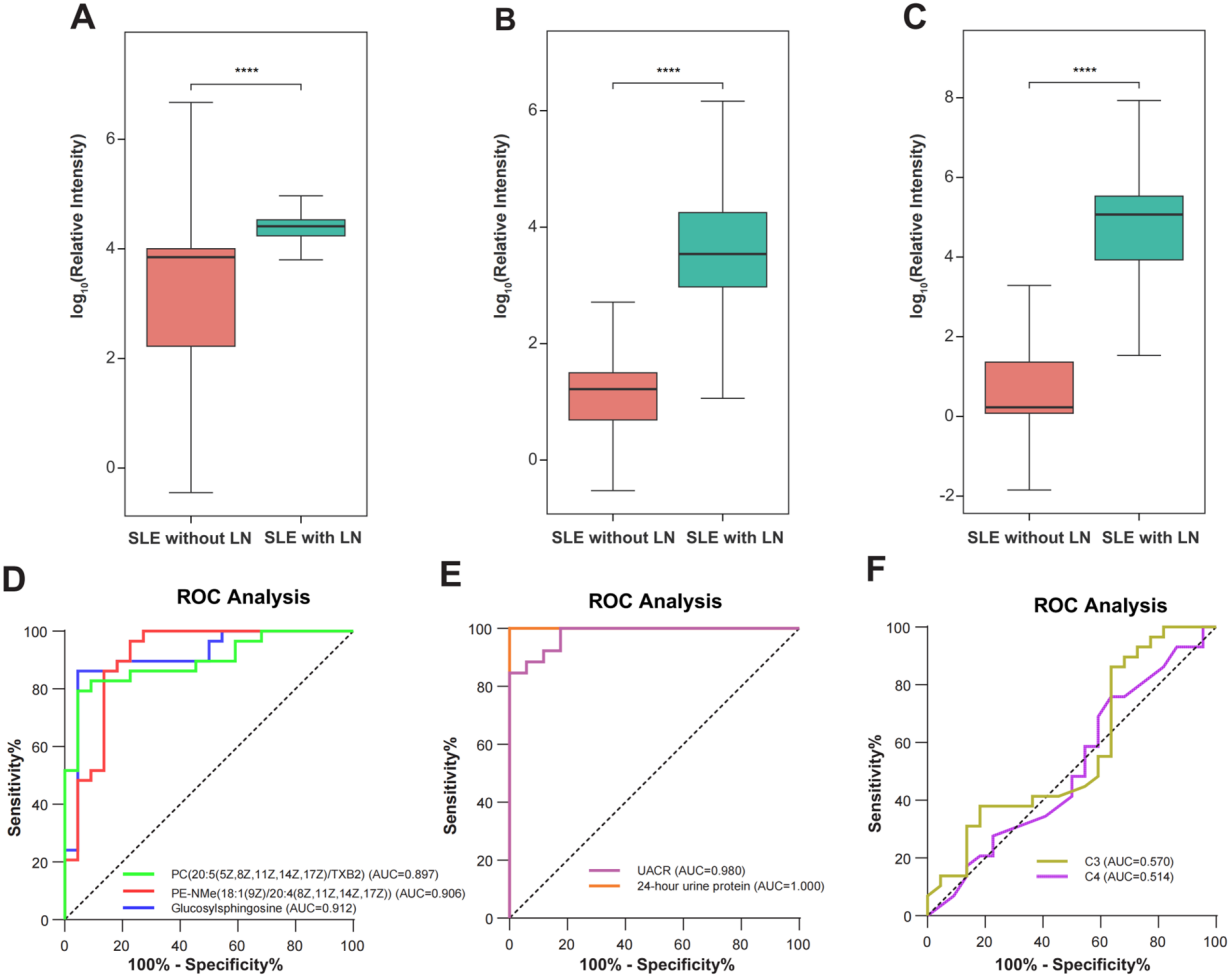
Metabolite biomarker distributions and receiver operating characteristic (ROC) curves. **(A-C)** Box plots illustrating the differential abundance of three potential biomarker metabolites in non-renal SLE (n=22) and SLE with LN (n=29) groups. The specific metabolites are: **(A)** Lyso-Gb1, **(B)** PE-NMe(18:1(9Z)/20:4(8Z,11Z,14Z,17Z)), and **(C)** PC(20:5(5Z,8Z,11Z,14Z,17Z)/TXB2). Statistical significance was determined by the Mann-Whitney U test, indicated by ****p < 0.0001. **(D)** ROC curves evaluating the diagnostic performance of the three metabolites in discriminating LN from non-renal SLE. The Area Under the Curve (AUC) values are: Glucosylsphingosine (AUC = 0.912, 95% CI: 0.825-0.999), PE-NMe (AUC = 0.906, 95% CI: 0.812-1.000), and PC (AUC = 0.897, 95% CI: 0.810-0.983). **(E-F)** ROC curves of conventional clinical indicators for LN diagnosis. **(E)** UACR (AUC = 0.980, 95% CI: 0.948-1.000) and 24-hour urine protein (AUC = 1.000, 95% CI: 0.825-1.000) show high discriminatory power. **(F)** C3 (AUC = 0.570, 95% CI: 0.406-0.735) and C4 (AUC = 0.514, 95% CI: 0.350-0.678) show limited diagnostic value.

Receiver operating characteristic (ROC) curve analyses were performed to evaluate the diagnostic performance of uEV metabolites for LN. The selected metabolites demonstrated robust discriminatory capacity (**Figure 7**), with detailed area under the curve (AUC) values provided in **Supplementary Table 4**. Among them, Glucosylsphingosine (Lyso-Gb1), PE-NMe(18:1(9Z)/20:4(8Z,11Z,14Z,17Z)), and PC(20:5(5Z,8Z,11Z,14Z,17Z)/TXB2) showed the highest diagnostic performance, achieving AUCs of 0.912, 0.906, and 0.897, respectively. To determine whether these uEV metabolites provide added diagnostic value beyond conventional clinical and laboratory markers, their discriminatory performance was systematically compared with established indicators of renal involvement and disease activity, including 24-hour urinary protein excretion, urine albumin-to-creatinine ratio (UACR), and complement levels (C3 and C4). As LN patients were defined and stratified according to 24-hour urinary protein, this parameter exhibited an expected near-perfect discriminatory performance (ROC AUC = 1.00), while UACR also demonstrated excellent performance (ROC AUC = 0.98). In contrast, complement C3 and C4 showed comparatively limited discriminatory ability (**Figure 8**). Notably, uEV metabolites not only effectively distinguished LN from non-LN patients but were also closely associated with disease activity and renal dysfunction, with these associations largely independent of proteinuria and complement levels. Collectively, these findings suggest that uEV metabolomic signatures capture metabolic and pathophysiological alterations not fully reflected by conventional proteinuria-based markers, thereby providing complementary molecular information for refined assessment and stratification of LN.

### Correlation analyses between the top three metabolites and key clinical and treatment-related variables

**Figure 9:**
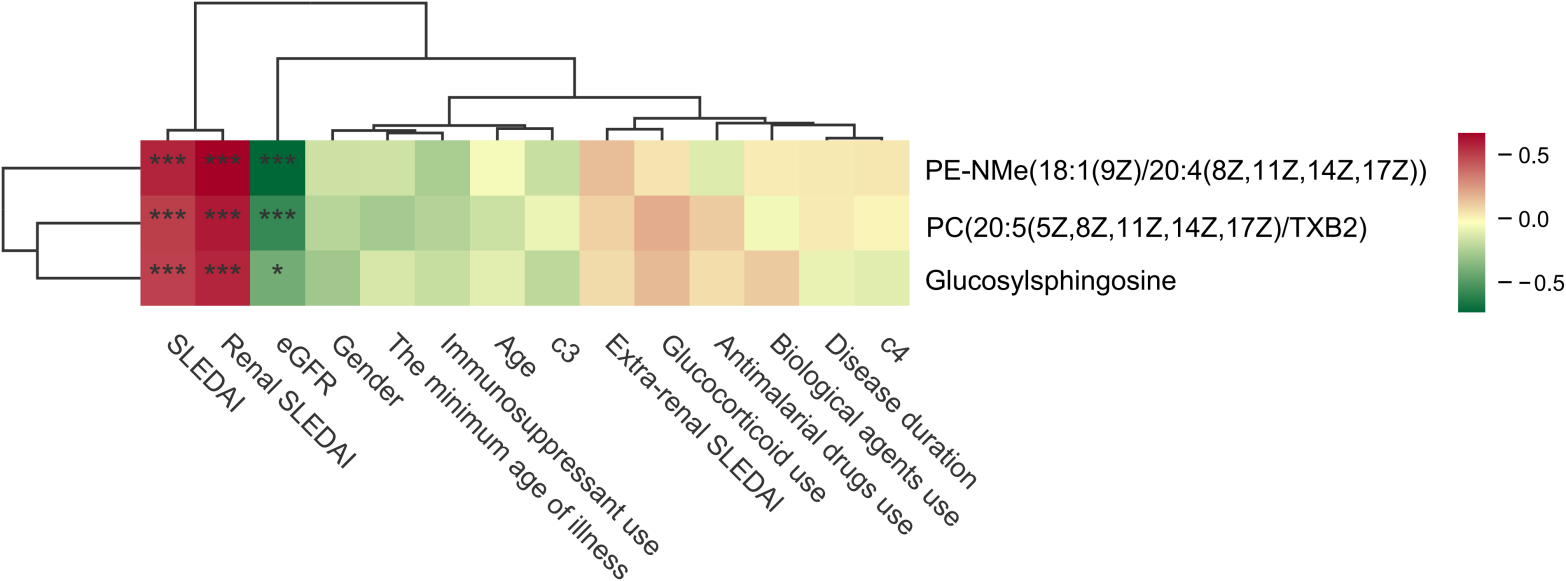
Correlation analyses between the top three metabolites and key clinical and treatment-related variables. The symbols “*” and “***” indicate statistical significance based on Spearman rank correlation analysis. Specifically, “*” represents P < 0.05 and “***” represents P < 0.001.

To further clarify whether the identified metabolites were primarily associated with disease activity rather than treatment exposure, we performed correlation analyses between the top three metabolites and key clinical and treatment-related variables (**Figure 9**). The analyses demonstrated that all three metabolites were positively correlated with disease activity indices, including SLEDAI and renal SLEDAI (rSLEDAI), and negatively correlated with estimated glomerular filtration rate (eGFR). In contrast, no significant correlations were observed between these metabolites and treatment-related variables, including corticosteroid use, immunosuppressive agents, or hydroxychloroquine, nor with other clinical parameters such as complement levels, age, or medication status. These results indicate that the top metabolites are more closely linked to disease activity and renal impairment rather than medication exposure, supporting their relevance as disease-associated metabolic signatures.

## Discussion

SLE is a chronic autoimmune disorder characterized by multi-organ involvement, among which LN represents one of the most severe manifestations with critical prognostic implications (27). Despite advances in clinical management, the early detection and dynamic monitoring of LN are hindered by the absence of reliable, non-invasive biomarkers; renal biopsy, while diagnostic, is invasive and unsuitable for frequent longitudinal assessment (28–30).

In this study, we conducted a comprehensive, high-resolution, untargeted LC–MS/MS metabolomic analysis of uEVs derived from the HC, SLE without LN, and SLE with LN groups. Collectively, these findings indicate that LN-associated uEV metabolomic signatures capture coordinated alterations in lipid metabolism, membrane remodeling, and inflammatory signaling within the kidney, supporting their biological relevance and translational potential as non-invasive biomarkers.

Machine learning–based feature selection highlighted pronounced metabolite alterations in uEVs from the SLE with LN group compared with those from the SLE without LN and HC groups. The random forest model identified ten metabolites with strong discriminatory capability, among which Glucosylsphingosine (Lyso-Gb1), PE-NMe(18:1(9Z)/20:4(8Z,11Z,14Z,17Z)), and PC(20:5(5Z,8Z,11Z,14Z,17Z)/TXB2) exhibited the highest diagnostic performance, with AUC values of 0.912, 0.906, and 0.897, respectively. Interestingly, several exogenous compounds, such as Asparagoside A and Ciguatoxin-3, were also identified, implying that dietary or environmental exposures may contribute to shaping LN-specific uEV metabolic profiles. The uEV metabolome of LN patients shows coordinated alterations in biologically meaningful pathways, including sphingolipid metabolism, phospholipid remodeling, and oxidative lipid signaling. Elevated Glucosylsphingosine reflects perturbed sphingolipid turnover potentially linked to podocyte and tubular stress, PE-NMe accumulation suggests reprogramming of phosphatidylethanolamine methylation associated with membrane remodeling, and oxidized PC species indicate lipid peroxidation within inflamed glomeruli. Additionally, detection of drug-related compounds such as hydroxychloroquine highlights the potential influence of therapeutic interventions on uEV metabolite composition, underscoring the need to distinguish disease-associated changes from treatment effects.

Moreover, an uncharacterized molecule, 4-(3-Methyl-5-oxo-4,5-dihydro-1H-pyrazol-1-yl)benzoic acid, demonstrated high predictive importance, further highlighting the exploratory and discovery potential of uEV metabolomics (31). After excluding exogenous/drug-derived peaks, several endogenous metabolites remained highly informative. The accumulation of Glucosylsphingosine (Lyso-Gb1) in LN uEVs indicates perturbation of sphingolipid metabolism and may reflect podocyte or tubular epithelial stress—findings consistent with prior reports of elevated glycosphingolipids in urine EVs from LN patients and the clinical utility of Lyso-Gb1 as a sensitive sphingolipid biomarker (31–33).

The elevation of PE-NMe, a monomethyl-phosphatidylethanolamine (PE) intermediate, indicates a reprogramming of phosphatidylethanolamine methylation (PEMT-associated) pathways during renal inflammation and membrane remodeling, thereby mechanistically linking dysregulated PE methylation to inflammatory lipid signaling (34). The detection of oxidized phosphatidylcholine species, such as PC(20:5/TXB2), likely reflects ongoing lipid peroxidation and aberrant arachidonic acid metabolism within inflamed glomeruli, aligning with experimental evidence demonstrating OxPC formation in renal ischemia–reperfusion and other inflammatory kidney injuries (35). Although PS(22:6-2OH/22:1) exhibited a relatively lower AUC, its well-established immunomodulatory functions are noteworthy. Increased levels of PS species may reflect impaired clearance of apoptotic bodies or altered immune complex processing—pathogenic mechanisms that are fundamental to the development of LN (36).

In summary, the integration of machine learning with uEV metabolomics provides a framework for characterizing renal metabolic alterations in LN and identifies candidate biomarkers that may support non-invasive and adjunctive diagnosis and monitoring. However, this study has several limitations. It is a single-center study with a moderate sample size, and residual confounding by disease activity, renal function indices (e.g., eGFR and proteinuria), and medication exposure (corticosteroids, immunosuppressive agents, hydroxychloroquine) cannot be fully excluded, which may influence uEV metabolite and lipid profiles. Only a subset of patients underwent contemporaneous renal biopsy, limiting correlation with histopathological class and activity, and urine samples were generally collected prior to biopsy, typically within one week. The use of a precipitation-based uEV isolation method may co-isolate non-EV components and affect detected metabolomic signatures, particularly lipid-related features. These limitations highlight the need for validation in independent, multi-center, and ethnically diverse cohorts using standardized and more stringent EV isolation strategies, as well as analytical frameworks that allow systematic adjustment or stratification by key clinical covariates. Finally, mechanistic studies and prospective, biopsy-synchronized cohorts—including stratification by LN class and activity—will be important to determine whether the identified metabolites act as causal mediators of LN pathogenesis or represent secondary consequences of renal injury. Future work should also emphasize longitudinal sampling, integration with single-cell and spatial omics, and functional perturbation assays to facilitate clinical translation of uEV-derived biomarkers (30).

## Data Availability

The metabolomics data of human urine presented in the study are deposited in the MetaboLights repository, accession number MTBLS13839.
