# Supplementary material for: Urinary extracellular vesicle metabolomic profiling reveals a distinct molecular signature for the non-invasive diagnosis of lupus nephritis
Source: Front Immunol. 2026 Feb 17;17:1741455. doi: 10.3389/fimmu.2026.1741455 (PMC12953380; doi:10.3389/fimmu.2026.1741455)
Supplement: Supplementary file 1 [file Table1.docx]

**Urinary Extracellular Vesicle Metabolomic Profiling Reveals a Distinct Molecular Signature for the Non-Invasive Diagnosis of Lupus Nephritis**

Nan Zhang^1+^, Ning Dong^1+^, Anran Xie^1+^, Wenjing Liu^1^, Adeel Khan^3^, Yanjing Rui^4*^, Ping Yang^1,2*^

^1^*Department of Laboratory Medicine, Nanjing Drum Tower Hospital, Affiliated Hospital of Medical School, Nanjing University, Nanjing, China*

*^2^State Key Laboratory of Pharmaceutical Biotechnology, Jiangsu Engineering Research Center for MicroRNA Biology and Biotechnology, NJU Advanced Institute of Life Sciences (NAILS), Nanjing University, Nanjing, China*

*^3^Department of Biotechnology, University of Science and Technology Bannu, 28100, Bannu, KP, Pakistan*

*^4^Department of Obstetrics and Gynecology, The Affiliated Drum Tower Hospital of Nanjing University Medical School, Nanjing, China*

**Correspondence:**
Ping Yang (pingyang@njglyy.com); Yanjing Rui (578499786@163.com).

**Supplementary Table 1: Significantly different metabolic pathways between SLE and HC.**

| **Num** | **First Category** | **Second Category** | **Pathway Desciption** | **PathwayID** | **Enrich**  **Factor** | **DA Score** | **Ratio**  **in study** | **Ratio in pop** | ***P* value** |
| --- | --- | --- | --- | --- | --- | --- | --- | --- | --- |
| 1 | Human Diseases | Drug resistance: antineoplastic | EGFR tyrosine kinase inhibitor resistance | map01521 | 0.5 | 0.1 | 1/101 | 2/4519 | 0.04421 |
| 2 | Organismal Systems | Circulatory system | Vascular smooth muscle contraction | map04270 | 0.125 | 0 | 2/101 | 16/4519 | 0.04842 |
| 2 | Human Diseases | Infectious disease: parasitic | Amoebiasis | map05146 | 0.153846 | 0 | 2/101 | 13/4519 | 0.03286 |
| 2 | Organismal Systems | Endocrine system | Regulation of lipolysis in adipocytes | map04923 | 0.142857 | 0 | 2/101 | 14/4519 | 0.03779 |
| 2 | Human Diseases | Cancer: overview | Choline metabolism in cancer | map05231 | 0.181818 | 0.04082 | 2/101 | 11/4519 | 0.02385 |
| 2 | Environmental Information Processing | Signal transduction | Sphingolipid signaling pathway | map04071 | 0.133333 | 0 | 2/101 | 15/4519 | 0.04298 |
| 2 | Environmental Information Processing | Signal transduction | HIF-1 signaling pathway | map04066 | 0.133333 | 0 | 2/101 | 15/4519 | 0.04298 |
| 2 | Organismal Systems | Nervous system | Dopaminergic synapse | map04728 | 0.166667 | 0 | 2/101 | 12/4519 | 0.02821 |
| 2 | Organismal Systems | Nervous system | Cholinergic synapse | map04725 | 0.166667 | 0 | 2/101 | 12/4519 | 0.02821 |
| 3 | Metabolism | Glycan biosynthesis and metabolism | Glycosylphosphatidylinositol (GPI)-anchor biosynthesis | map00563 | 0.285714 | -0.02222 | 2/101 | 7/4519 | 0.009654 |
| 3 | Metabolism | Metabolism of cofactors and vitamins | Pantothenate and CoA biosynthesis | map00770 | 0.1 | -0.2727 | 3/101 | 30/4519 | 0.02842 |
| 3 | Human Diseases | Infectious disease: viral | Kaposi sarcoma-associated herpesvirus infection | map05167 | 0.4 | -0.01852 | 2/101 | 5/4519 | 0.004734 |
| 4 | Metabolism | Metabolism of other amino acids | beta-Alanine metabolism | map00410 | 0.125 | -0.2857 | 4/101 | 32/4519 | 0.005227 |
| 4 | Metabolism | Global and overview maps | Nucleotide metabolism | map01232 | 0.068966 | -0.08696 | 4/101 | 58/4519 | 0.03974 |
| 4 | Metabolism | Amino acid metabolism | Glycine, serine and threonine metabolism | map00260 | 0.083333 | 0 | 4/101 | 48/4519 | 0.02156 |
| 5 | Metabolism | Amino acid metabolism | Lysine degradation | map00310 | 0.089286 | -0.12 | 5/101 | 56/4519 | 0.007822 |
| 5 | Metabolism | Xenobiotics biodegradation and metabolism | Drug metabolism - cytochrome P450 | map00982 | 0.057471 | -0.05 | 5/101 | 87/4519 | 0.04413 |
| 6 | Metabolism | Biosynthesis of other secondary metabolites | Caffeine metabolism | map00232 | 0.272727 | -0.4 | 6/101 | 22/4519 | 6.00E-06 |
| 6 | Metabolism | Amino acid metabolism | Tryptophan metabolism | map00380 | 0.072289 | 0 | 6/101 | 83/4519 | 0.01011 |
| 6 | Metabolism | Lipid metabolism | Glycerophospholipid metabolism | map00564 | 0.089286 | -0.02797 | 5/101 | 56/4519 | 0.007822 |
| 7 | Environmental Information Processing | Membrane transport | ABC transporters | map02010 | 0.050725 | -0.125 | 7/101 | 138/4519 | 0.03375 |
| 11 | Organismal Systems | Digestive system | Bile secretion | map04976 | 0.072165 | -0.225 | 7/101 | 97/4519 | 0.005607 |

**Supplementary Table 2: Significantly different metabolic pathways between SLE with LN and SLE without LN.**

| **Num** | **First Category** | **Second Category** | **Pathway Desciption** | **Pathway**  **ID** | **Enrich Factor** | **DA Score** | **Ratio**  **in study** | **Ratio in pop** | ***P* value** |
| --- | --- | --- | --- | --- | --- | --- | --- | --- | --- |
| 1 | Human Diseases | Drug resistance: antineoplastic | EGFR tyrosine kinase inhibitor resistance | map01521 | 0.5 | 0.1 | 1/48 | 2/4519 | 0.02113 |
| 1 | Cellular Processes | Transport and catabolism | Autophagy - other | map04136 | 0.333333 | 0.02273 | 1/48 | 3/4519 | 0.03154 |
| 1 | Organismal Systems | Immune system | Th1 and Th2 cell differentiation | map04658 | 0.333333 | 0.1 | 1/48 | 3/4519 | 0.03154 |
| 1 | Human Diseases | Infectious disease: bacterial | Pathogenic Escherichia coli infection | map05130 | 0.333333 | 0.02273 | 1/48 | 3/4519 | 0.03154 |
| 1 | Environmental Information Processing | Signal transduction | NF-kappa B signaling pathway | map04064 | 0.333333 | 0.1 | 1/48 | 3/4519 | 0.03154 |
| 1 | Organismal Systems | Immune system | T cell receptor signaling pathway | map04660 | 0.25 | 0.1 | 1/48 | 4/4519 | 0.04183 |
| 1 | Organismal Systems | Immune system | Natural killer cell mediated cytotoxicity | map04650 | 0.25 | 0.1 | 1/48 | 4/4519 | 0.04183 |
| 1 | Organismal Systems | Immune system | Th17 cell differentiation | map04659 | 0.25 | 0.1 | 1/48 | 4/4519 | 0.04183 |
| 1 | Human Diseases | Cancer: overview | PD-L1 expression and PD-1 checkpoint pathway in cancer | map05235 | 0.25 | 0.1 | 1/48 | 4/4519 | 0.04183 |
| 1 | Human Diseases | Cancer: specific types | Glioma | map05214 | 0.25 | 0.1 | 1/48 | 4/4519 | 0.04183 |
| 1 | Environmental Information Processing | Signal transduction | ErbB signaling pathway | map04012 | 0.25 | 0.1 | 1/48 | 4/4519 | 0.04183 |
| 1 | Organismal Systems | Immune system | B cell receptor signaling pathway | map04662 | 0.25 | 0.1 | 1/48 | 4/4519 | 0.04183 |
| 1 | Organismal Systems | Endocrine system | Growth hormone synthesis, secretion and action | map04935 | 0.25 | 0.1 | 1/48 | 4/4519 | 0.04183 |
| 2 | Human Diseases | Infectious disease: viral | Kaposi sarcoma-associated herpesvirus infection | map05167 | 0.4 | 0.03704 | 2/48 | 5/4519 | 0.001083 |
| 2 | Human Diseases | Infectious disease: parasitic | African trypanosomiasis | map05143 | 0.25 | 0.1818 | 2/48 | 8/4519 | 0.00297 |
| 2 | Organismal Systems | Immune system | Platelet activation | map04611 | 0.142857 | 0.1667 | 2/48 | 14/4519 | 0.009269 |
| 2 | Organismal Systems | Nervous system | Retrograde endocannabinoid signaling | map04723 | 0.105263 | 0.02564 | 2/48 | 19/4519 | 0.01684 |
| 2 | Metabolism | Metabolism of cofactors and vitamins | Riboflavin metabolism | map00740 | 0.083333 | 0 | 2/48 | 24/4519 | 0.02628 |
| 3 | Organismal Systems | Nervous system | Serotonergic synapse | map04726 | 0.071429 | 0.1364 | 3/48 | 42/4519 | 0.00966 |
| 3 | Metabolism | Amino acid metabolism | Glycine, serine and threonine metabolism | map00260 | 0.0625 | 0.02381 | 3/48 | 48/4519 | 0.01392 |
| 3 | Metabolism | Amino acid metabolism | Lysine degradation | map00310 | 0.053571 | 0.04 | 3/48 | 56/4519 | 0.02104 |
| 4 | Human Diseases | Cancer: overview | Choline metabolism in cancer | map05231 | 0.181818 | 0.08163 | 2/48 | 11/4519 | 0.005717 |
| 4 | Organismal Systems | Digestive system | Bile secretion | map04976 | 0.041237 | 0.05 | 4/48 | 97/4519 | 0.01887 |
| 7 | Metabolism | Lipid metabolism | Glycerophospholipid metabolism | map00564 | 0.089286 | 0.04895 | 5/48 | 56/4519 | 0.000278 |

**Supplementary Table 3: Importance values of differential metabolites between SLE with LN and SLE without LN in the random-forest model.**

| **Feature** | **Metab ID** | **Importance** |
| --- | --- | --- |
| Asparagoside A | metab_19773 | 0.115331575 |
| Ciguatoxin-3 | metab_15134 | 0.071956313 |
| 4-(3-Methyl-5-oxo-4,5-dihydro-1H-pyrazol-1-yl)benzoic acid | metab_31368 | 0.06004255 |
| PE-NMe(18:1(9Z)/20:4(8Z,11Z,14Z,17Z)) | metab_33940 | 0.057493874 |
| Glucosylsphingosine | metab_17707 | 0.051329312 |
| PS(22:6(5Z,8E,10Z,13Z,15E,19Z)-2OH(7S, 17S)/22:1(13Z)) | metab_17155 | 0.051303745 |
| Gingerglycolipid B | metab_19770 | 0.046077965 |
| Herbimycin a | metab_18820 | 0.040368397 |
| Aspirin | metab_16575 | 0.039069816 |
| N6-(Delta2-Isopentenyl)-adenine | metab_29749 | 0.034443817 |
| (4E)-3-Hydroxyhex-4-enoylcarnitine | metab_10278 | 0.03298006 |
| DG(i-15:0/0:0/18:1(9Z)-O(12,13)) | metab_26865 | 0.028839972 |
| Triflusal | metab_21834 | 0.028426596 |
| PC(20:5(5Z,8Z,11Z,14Z,17Z)/TXB2) | metab_15348 | 0.026458913 |
| 6,15-Diketo-13,14-dihydro-PGF1α | metab_21498 | 0.02514976 |
| Polysorbate 20 | metab_8764 | 0.024362961 |
| SM(d16:2(4E,8Z)/PGE1) | metab_15536 | 0.02325286 |
| 1-[(3R,4R,5R)-3-Fluoro-3,4-dihydroxy-5-(1-hydroxyethyl)oxolan-2-yl]pyrimidine-2,4-dione | metab_12407 | 0.022718944 |
| Americine | metab_15139 | 0.021694578 |
| Nipradilol | metab_21313 | 0.020985486 |
| (4R,6S)-6-[(E)-2-[2-(4-Fluoro-3-methylphenyl)-4,6-dimethylphenyl]ethenyl]-4-hydroxyoxan-2-one | metab_21224 | 0.020547633 |
| Asparaginyl-Valine | metab_25293 | 0.018247875 |
| Oxaprozin glucuronide | metab_29374 | 0.017911534 |
| Histidyllysine | metab_28313 | 0.013970588 |
| Androsta-1,4-diene-17-carboxylicacid, 17-[(ethoxycarbonyl)oxy]-11-hydroxy-3-oxo-, fluoromethyl ester, (11b,17a)- | metab_15002 | 0.013424128 |
| Imazamethabenz-methyl | metab_9419 | 0.013412748 |
| Acetaminophen | metab_14726 | 0.013308682 |
| 1-Linoleoyl-sn-Glycero-3-Phosphocholine | metab_27206 | 0.012775021 |
| Hydroxydehydro Nifedipine Carboxylate | metab_4952 | 0.012711592 |
| Pinobanksin 5-[galactosyl-(1->4)-glucoside] | metab_32667 | 0.011178024 |
| 6-Hydroxynon-6-enoylcarnitine | metab_15195 | 0.00681173 |
| Undec-3-enedioylcarnitine | metab_31534 | 0.006654623 |
| Verbenalin | metab_14949 | 0.00660639 |
| Cyclo(Arg-Gly-Asp-D-Phe-Val) | metab_22835 | 0.005360272 |
| 2,4-Hexadien-1-ol | metab_31471 | 0.004791664 |

**Supplementary Table 4: AUC values of differential metabolites of lupus nephritis.**

| **Metabolite** | **Metab ID** | **AUC** | **95%CI** |
| --- | --- | --- | --- |
| Glucosylsphingosine | metab_17707 | 0.9122 | [0.8254, 0.999] |
| PE-NMe(18:1(9Z)/20:4(8Z,11Z,14Z,17Z)) | metab_33940 | 0.906 | [0.8121, 0.9998] |
| PC(20:5(5Z,8Z,11Z,14Z,17Z)/TXB2) | metab_15348 | 0.8966 | [0.8098, 0.9833] |
| (4E)-3-Hydroxyhex-4-enoylcarnitine | metab_10278 | 0.8918 | [0.7939, 0.9898] |
| DG(i-15:0/0:0/18:1(9Z)-O(12,13)) | metab_26865 | 0.8715 | [0.7642, 0.9788] |
| 4-(3-Methyl-5-oxo-4,5-dihydro-1H-pyrazol-1-yl)benzoic acid | metab_31368 | 0.8699 | [0.7722, 0.9676] |
| PS(22:6(5Z,8E,10Z,13Z,15E,19Z)-2OH(7S, 17S)/22:1(13Z)) | metab_17155 | 0.8527 | [0.7275, 0.9778] |
| SM(d16:2(4E,8Z)/PGE1) | metab_15536 | 0.8292 | [0.6989, 0.9594] |
| N6-(Delta2-Isopentenyl)-adenine | metab_29749 | 0.8072 | [0.6774, 0.937] |
| 6,15-Diketo-13,14-dihydro-PGF1alpha | metab_21498 | 0.8056 | [0.6787, 0.9326] |
